# Characterisation of systemic dissemination of nonreplicating adenoviral vectors from tumours in local gene delivery

**DOI:** 10.1038/sj.bjc.6602494

**Published:** 2005-04-05

**Authors:** Y Wang, Z Yang, S Liu, T Kon, A Krol, C-Y Li, F Yuan

**Affiliations:** 1 Department of Biomedical Engineering, Duke University, Durham, NC 27708, USA; 2 Department of Radiation Oncology, Duke University Medical Center, Durham, NC 27710, USA

**Keywords:** gene delivery, virus dissemination, intratumoral infusion

## Abstract

Systemic virus dissemination is a potential problem during local gene delivery in solid tumours. However, the kinetics and pathways of the dissemination have not been well characterised during the first 24 h after the infusion is started. To this end, we infused adenoviral vectors for luciferase or enhanced green fluorescence protein into three different tumour models in mice. During and/or after the infusion, we determined the amount of adenoviruses in the tumour, blood, and liver, and examined the transgene expression in the liver, lung, blood, and tumour. In addition, we intravenously injected tumour cells expressing luciferase and examined the biodistribution of these cells in the body. We observed transgene expression in the liver and tumour at 24 h after the infusion, but could not detect transgene expression in the blood and lung. The peak concentration of viral vectors in the plasma occurred during the intratumoral infusion. At 10 min after the infusion, few viral vectors remained in the blood and the ratio of copy numbers of adenoviruses between liver and tumour was >2 in 80% and ⩾10 in 40% of the mice. Most tumour cells injected intravenously accumulated in the lung within the first 24 h. Taken together, these data indicated that systemic virus dissemination occurred mainly during the first 10 min after the intratumoral infusion was started, and that the dissemination was due to infusion-induced convective transport of viral vectors into leaky tumour microvessels.

Gene therapy represents a new paradigm to treat cancer at the genetic level (Ledley, 1996; Anderson, 1998). Its success relies on the ability to deliver sufficient genetic materials into target cells. The most widely used vectors for gene delivery are genetically engineered viruses, because they, in the long-term evolution of nature, are equipped with specific machineries that facilitate DNA transport into the nucleus of cells (Baranowski *et al*, 2001). However, a main drawback of viral vectors is the toxicity caused by undesirable acute immune response to viral proteins (Brown and Lillicrap, 2002; Bowers *et al*, 2003; Chen *et al*, 2003; Jooss and Chirmule, 2003; Lowenstein, 2003). Moreover, proteins from transgene expression may also exert adverse effects on normal cells either directly or indirectly through conversion of prodrugs to cytotoxic agents (Freeman *et al*, 1993; Rosenfeld *et al*, 1995; Alvarez and Curiel, 1997; Pope *et al*, 1997; Chada *et al*, 2003). Both toxicity and efficacy of gene therapy increase with the dose of treatment. Therefore, the goal of research in gene delivery is to improve the efficacy/toxicity ratio (Crystal, 1995; Alemany *et al*, 2000a).

The improvement can be achieved through increasing the specificity of either vector delivery or transgene expression in tumour cells. One way to increase the delivery specificity is to physicochemically or genetically modify viral surface proteins so that the vectors will target specific biomarkers in tumours (Douglas *et al*, 1996; Hall *et al*, 1997). However, this approach has its limitations because such biomarkers may exist in normal tissues and distribute heterogeneously in tumours. To reduce transgene expression in normal tissues, unique promoters have been integrated into viral vectors. These promoters can be activated only by specific stimuli in tumours that are either endogenous or applied externally (Vaupel *et al*, 1989; Gossen and Bujard, 1992; Garver *et al*, 1994; Harris *et al*, 1994; Hallahan *et al*, 1995; Kumagai *et al*, 1996; Jaggar *et al*, 1997; Jenster *et al*, 1997; Blackburn *et al*, 1998; Chung *et al*, 1999; Abdul-Ghani *et al*, 2000; Huang *et al*, 2000; Koshikawa *et al*, 2000; Pollock *et al*, 2000; Spitz *et al*, 2000). However, the control of transgene expression will not reduce the acute toxicity in normal tissues caused by viral proteins.

Intratumoral infusion is another strategy for improving the efficacy/toxicity ratio in viral gene therapy. It has been widely used in cancer clinical trials. Researchers often assume that viral vectors and transgene expression are confined to solid tumours after the infusion, thereby causing minimal toxicity in normal tissues (Lu *et al*, 1999; Paielli *et al*, 2000). On the other hand, researchers in some studies have observed that transgene expression in normal tissues can be as strong as that in tumours (Toloza *et al*, 1996; Bramson *et al*, 1997; Nasu *et al*, 1999; Tjuvajev *et al*, 1999; Lohr *et al*, 2001; Wang *et al*, 2003), that adenoviral vectors are present in systemic circulation of patients in clinical trials (DeWeese *et al*, 2001; Nemunaitis *et al*, 2001), and that some animals die within a short period after intratumoral infusion (Toloza *et al*, 1996; Tjuvajev *et al*, 1999). These observations indicate that viral vectors can escape from tumours during and/or after intratumoral infusion. Thus, the improvement in efficacy/toxicity ratio via the intratumoral infusion is likely to be far from optimal.

Although virus dissemination has been observed in preclinical and clinical studies, its kinetics and pathways are still controversial. Therefore, we characterised the biodistributions of adenoviral vectors and transgene expression in the tumour, liver, blood, and lung at different time points after the infusion was started. We observed that the systemic virus dissemination occurred mainly within the first 10 min. The amount of viruses disseminated into the liver could be one order of magnitude higher than that retained in the tumour, and the mechanism of dissemination was the infusion-induced convective transport of viral vectors into leaky tumour microvessels.

## MATERIALS AND METHODS

### Adenoviral vectors

The Ad5-based recombinant system was used to produce the nonreplicating adenoviruses: AdCMVEGFP and AdCMVLuc encoding enhanced green fluorescence protein (EGFP) and luciferase, respectively. The cDNA for EGFP or luciferase was inserted into the E1 region of the adenovirus and the transgene expression was driven by the cytomegalovirus (CMV) promoter. Adenoviruses were propagated in 293 cells (ATCC, Manassas, VA, USA), harvested at 48 h after infection, and purified by the method of cesium chloride gradient centrifugation according to a standard protocol (Graham and Prevec, 1995). The viral vectors were stored in 10% glycerol at −82°C.

### Tumour cell lines

Three tumour cell lines (4T1, B16.F10, and IMR-23) were used in the study. 4T1 is a murine mammary carcinoma; B16.F10 is a metastatic subline of B16 murine melanoma; IMR-23 is a human neuroblastoma. All tumour cells were cultured in DMEM supplemented with 10% newborn bovine serum (Hyclone, Logan, UT, USA) and 1% penicillin/streptomycin (Gibco/Life Technologies, Grand Island, NY, USA) at 37°C with 5% CO_2_ and 95% air. For tumour implantation in mice, the cells were removed from culture flasks with 0.25% trypsin/EDTA and rinsed twice in PBS.

### Animals

Female Balb/c and C57BL/6 mice, 4–6 weeks old, were ordered from Charles River Laboratory (Wilmington, MA, USA) for the implantation of 4T1 and B16.F10 tumours, respectively. Female Balb/c nude mice at the same age as Balb/c mice were ordered from the National Cancer Institute (Bethesda, MD, USA) for the implantation of IMR-23 tumour. For tumour cell implantation, mice were first anesthetised with an intraperitoneal injection (0.15 ml) of a mixture of ketamine and xylazine (80 mg ketamine and 10 mg xylazine per kg body weight). Then, one million tumour cells in 50 *μ*l PBS were injected subcutaneously into the right hind limb of mice. After implantation, the mice were placed on a warm pad until they woke up. All subcutaneous tumours were ready for experiments when they reached 5–8 mm in diameter. After experiments, fully anaesthetised mice were killed with cervical dislocation. The use of animals and all experimental procedures involving animals in this study had been approved by the Duke University Institutional Animal Care and Use Committee. These procedures were also consistent with the United Kingdom Co-ordinating Committee on Cancer Research (UKCCCR) Guidelines (UKCCCR, 1998).

### Intratumoral infusion

Mice were anesthetised with an intraperitoneal injection (0.15 ml) of a mixture of ketamine and xylazine (80 mg ketamine and 10 mg xylazine per kg body weight). The tip of a 30-gauge needle was carefully placed near the centre of tumours by controlling the depth of needle insertion relative to the tumour size measured by a caliper. Virus suspension was infused into tumours via the needle mounted on a syringe pump (model 22, Harvard Apparatus Co., Cambridge, MA, USA). The volume of infusion was fixed at 50 *μ*l although the dose of viruses varied with experiments as indicated below. The infusion rate was chosen to be 1 *μ*l s^−1^ based on a preliminary study in which we infused 50 *μ*l of a solution of Evans blue dye or a suspension of an adenoviral vector for LacZ, AdCMVLacZ, into 4T1 tumours and observed that the distribution volume of Evans blue or *β*-galactosidase in tumours was the highest when the infusion rate was 1 *μ*l s^−1^.

### Luciferase and EGFP expression in different organs/tissues

At 24 h after intratumoral infusion of AdCMVLuc or AdCMVEGFP (2.0 × 10^8^ plaque forming units (pfu)), mice were anesthetised again with the same method as described above and transgene expression in tumours was examined. For luciferase analysis, 50 *μ*l of aqueous D-luciferin solution was injected intraperitoneally into the mice placed on a warm pad at 20 min before the examination of bioluminescence in the body. The bioluminescence was recorded by the *In Vivo* Imaging System (IVIS) (Xenogen Corp., Alameda, CA, USA), with an exposure time of 30 s (Wang *et al*, 2003) and the bioluminescence intensity was represented by pseudo-colours as indicated by a colour bar. The final images were generated by superimposing the pseudo-colour bioluminescence images on the reference images. For EGFP analysis, tissue samples from the blood, lung, liver, and tumour were harvested. Except the blood, tissue samples were sectioned into thin slices (300 *μ*m thick), using a Vibratome (Model 3000; Technical Products International, St Louis, MO, USA). Enhanced green fluorescence protein expression in the blood and sliced tissues was examined under a confocal laser-scanning microscope (LSM 510, Carl Zeiss, Thornwood, NY, USA).

In a separate experiment, we injected 10^6^ luciferase-expressing 4T1 cells in a 100-*μ*l suspension into the tail vein of Balb/c mice anesthetised with the same method as described above. At both 10 min and 24 h after the cell injection, luciferase expression in the body was examined using the same method as described above.

### Qualitative analysis of adenoviruses in the blood

At predetermined time points (25 s, 50 s, 5 min, 10 min, 2, and 24 h) after starting the infusion of AdCMVEGFP (3.0 × 10^8^ pfu) into 4T1 tumours, blood samples (approximately 20 *μ*l in each sample) from anesthetised mice were collected through the orbital sinus with a heparinised capillary, stored at room temperature for 10 min, and centrifuged at 10 000 **g** for 3 min to separate the plasma from blood cells. The plasma and cell suspension were then exposed to four cycles of thawing (37°C) and freezing (−196°C in liquid nitrogen) treatment. In all, 1 *μ*l of plasma solution or suspension of lysed blood cells was added into 96-well plates with 70–80% confluent 293 cells. After 24 h, EGFP expression in these cells was examined under a fluorescence microscope (Axiovert 100, Carl Zeiss).

### Quantitative analysis of adenovirus dissemination in liver and tumours

At 10 min after the infusion of AdCMVEGFP (3.0 × 10^8^ pfu) into 4T1 tumors, mice were killed with cervical dislocation and the liver and tumours were immediately harvested, frozen in liquid nitrogen, and stored at −82°C. For viral DNA analysis, the tissue samples were immersed in liquid nitrogen and ground into fine tissue powder in a mortar. Viral DNA was isolated from the tissue powder using a DNeasy Tissue kit (Qiagen, Clarita, CA, USA). The DNeasy mini column was eluted twice with 200 *μ*l of Tris buffer (10 mM, pH=8.5) to obtain viral DNA solution, in which Ad E4 copy number was measured with the Smart Cycler System (Cepheid, Sunnyvale, CA, USA). In the real-time PCR analysis, the primers and probes were purchased from Sigma-Genosis (Woodlands, TX USA). The forward primer sequence for Ad E4 region was: 5′-TGACACGCATACTCGGAGCTA-3′: 34 885–34 905; the reverse primer sequence was: 5′-TTTGAGCAGCACCTTGCATT-3′: 34 977–34 958; the fluorogenic probe sequence was: [DFAM]CGCCGCCCATGCAACAAGCTT[DBH1]: 34 930–34 951 (Adachi *et al*, 2001). Each PCR reaction was performed in duplicate in a 25-*μ*l reaction mixture containing 0.15 *μ*l of AmpliTaq Gold DNA polymerase (5 U *μ*l^−1^) (Applied Biosystems, Foster City, CA USA), 300 nM each primer, and 200 nM fluorogenic probe. The amplification consisted of an initial heating at 95°C for 10 min, followed by 40 cycles at 95°C for 15 s and 60°C for 45 s. The standard curve was generated by using serial dilutions of pFG140 plasmid DNA (Microbix Biosystems Inc., Ontario, Canada) in the genomic liver DNA solution.

## RESULTS

### Systemic AdCMVLuc dissemination from tumours

**Figure 1:**
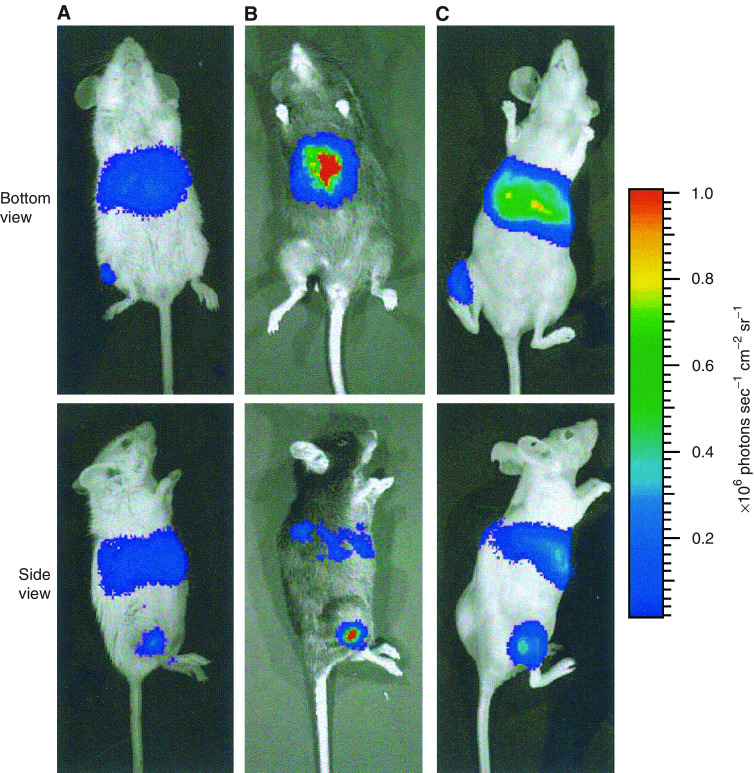
Typical images of luciferase distribution at 24 h after AdCMVLuc infusion into different tumours: (**A**) 4T1 in a Balb/c mouse, (**B**) B16.F10 in a C57BL/6 mouse, and (**C**) IMR-23 in a Balb/c nude mouse. The dose of infusion was 2.0 × 10^8^ pfu tumour^−1^.

To demonstrate the systemic dissemination of viral vectors, AdCMVLuc suspension was infused into two murine tumour models, 4T1 and B16.F10, respectively, and one human tumour xenograft, IMR-23. Bleeding was observed in many tumours immediately after the removal of infusion needle but the amount of bleeding was much less than the infused volume (i.e. 50 *μ*l). At 24 h after infusion, strong bioluminescence, an indication of luciferase expression, was observed in the liver and tumour in all animal models (see Figure 1). The bioluminescence from the liver indicated that AdCMVLuc had escaped from tumours in local gene delivery, because luciferase is an exogenous and nonsecretable protein. It should be retained in transfected, viable cells.

### Kinetics of adenovirus dissemination

**Figure 2:**
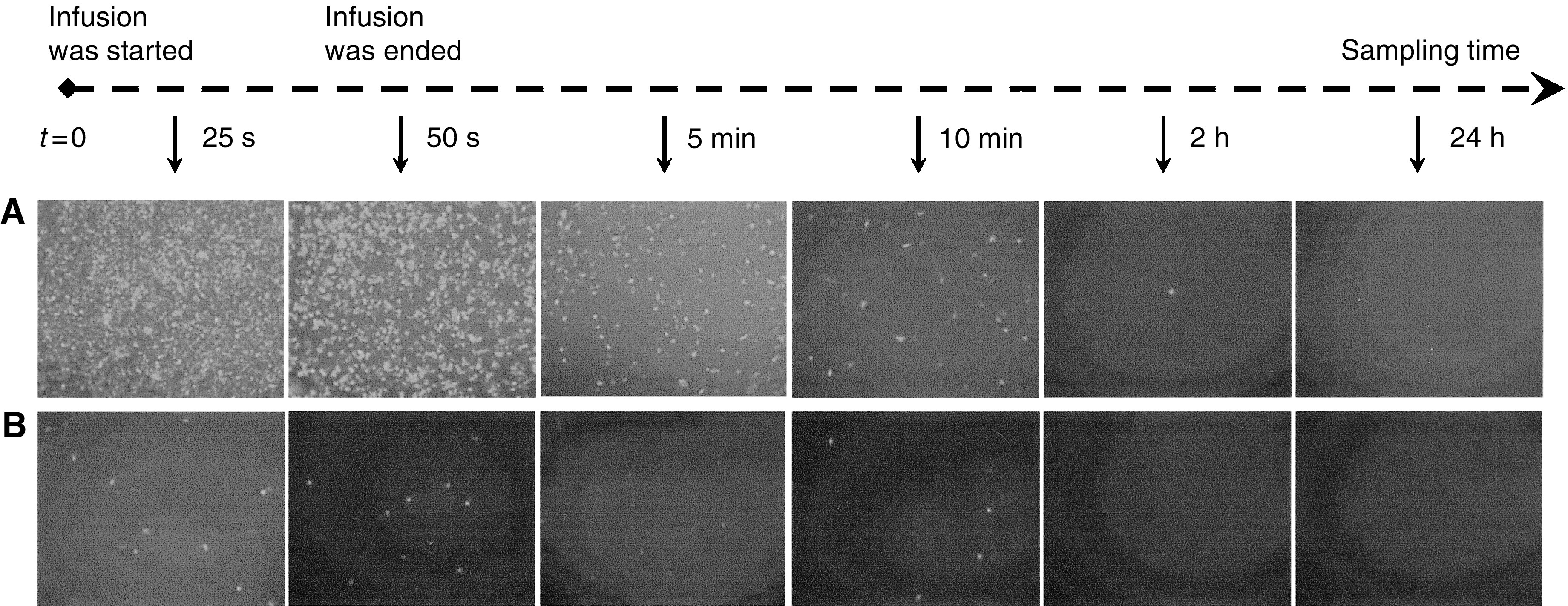
EGFP expression in 293 cells transfected by AdCMVEGFP that was isolated from (**A**) the plasma and (**B**) the lysed blood cells in mice. The blood samples were collected at different time points after the intratumoral infusion of AdCMVEGFP was started. The dose of infusion was 3.0 × 10^8^ pfu tumour^−1^. The infusion was performed at 1 *μ*l s^−1^ over a 50-s period.

The plasma from blood samples at different time points, both during and after the intratumoral infusion of AdCMVEGFP, contained the viral vectors that could transfect 293 cells *in vitro* (see Figure 2A). Qualitatively, the amount of AdCMVEGFP in the plasma reached the peak at the end of intratumoral infusion and then decreased quickly with time. It was significantly lower than the peak value at 10 min and nearly undetectable at 2 h after the infusion. Cells from blood samples contained only a few AdCMVEGFP that could transfect 293 cells *in vitro* (see Figure 2B), indicating that the virus dissemination was not mediated by transfected tumour or blood-borne cells. To our best knowledge, this was the first direct evidence that demonstrated the adenovirus dissemination during intratumoral infusion.

### Role of tumour or blood-borne cells in virus dissemination

**Figure 3:**
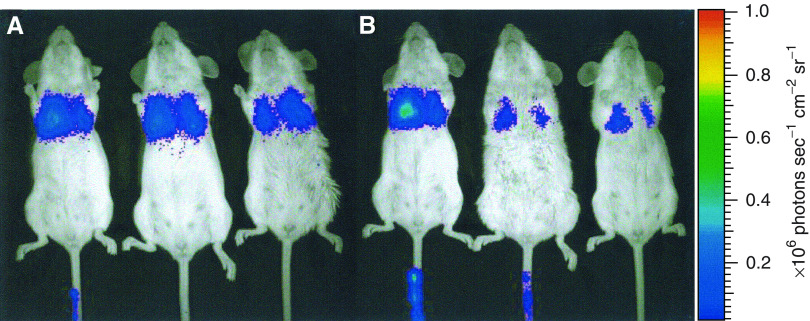
Typical images of luciferase distribution in three mice at (**A**) 10 min and (**B**) 24 h after intravenous injection of 4T1 cells expressing luciferase. The dose of injection was 10^6^ cells per mouse.

**Figure 4:**
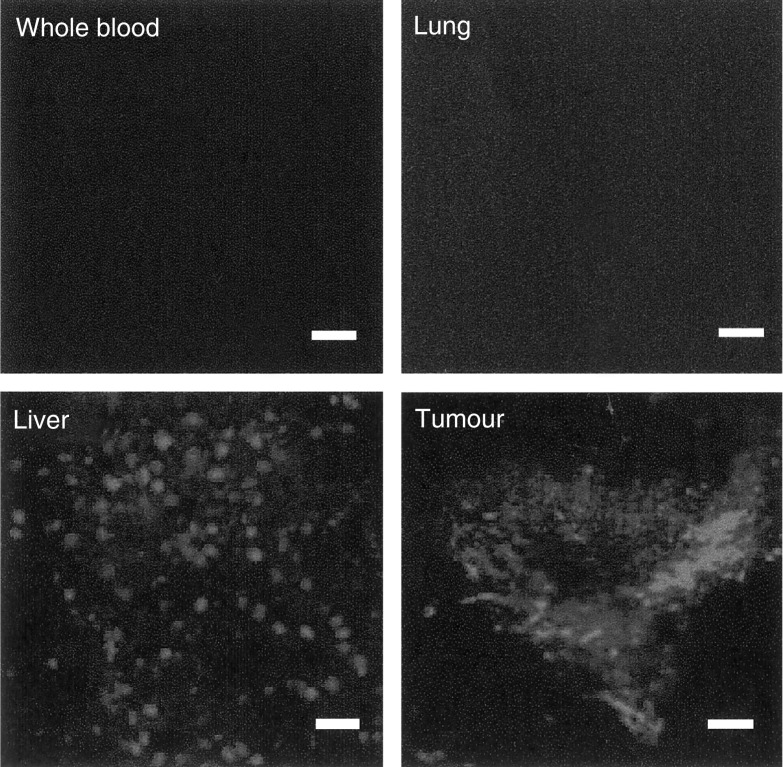
Typical images of EGFP distribution in different tissues at 24 h after the infusion of AdCMVEGFP into 4T1 tumours. Bar=100 *μ*m. The dose of infusion was 3.0 × 10^8^ pfu tumour^−1^.

To further investigate the role of tumour or blood-borne cells in virus dissemination, we performed two additional experiments. Firstly, we infused luciferase-expressing cells into the tail vein of mice, and observed that nearly all infused cells accumulated in the lung (Figure 3). Secondly, we infused AdCMVEGFP vectors into 4T1 tumours and examined EGFP expression in the liver, tumour, lung, and blood at 24 h after the infusion. We observed EGFP expression in liver and tumour tissues but could not detect EGFP-expressing cells in the lung and blood (Figure 4). These results confirmed it again that the virus dissemination was not mediated by transfected tumour or blood-borne cells.

### Quantitative measurement of adenoviruses in liver and tumour tissues

To quantitatively understand the significance of systemic adenovirus dissemination at early time points, we quantified the copy numbers of adenoviruses in liver and tumour tissues at 10 min after the intratumoral infusion of AdCMVEGFP. We chose this time point for three reasons. Firstly, data in the literature show that adenoviruses have a plasma half-life of ∼2 min (Worgall *et al*, 1997; Alemany *et al*, 2000b) (see also Figure 2A in this study). Thus, approximately 97% of disseminated viruses have been cleared from the blood at 10 min. Secondly, the clearance in mice is mainly through the liver (Herz and Gerard, 1993; Hackett *et al*, 2000). Finally, the amount of active adenoviral vectors in the liver decreases with time (Worgall *et al*, 1997). As a result, the copy numbers of adenoviruses in the liver at 10 min after the infusion provided the best estimate of the total amount of adenoviruses disseminated from the tumour.

**Figure 5:**
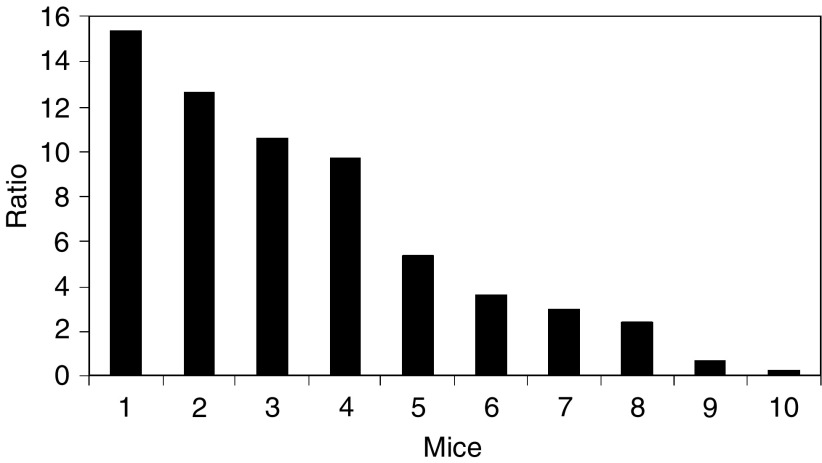
Ratio of virus copy numbers between liver and tumour at 10 min after the infusion of AdCMVEGFP into 4T1 tumours implanted in 10 different mice.

The ratio of the copy numbers of adenoviruses between liver and tumour is shown in Figure 5. The minimal value was 0.2; eight out of 10 mice had a ratio greater than 2; and four mice had a ratio close to or greater than 10. These data demonstrated that a significant amount of adenoviruses had disseminated from tumours within 10 min after the intratumoral infusion.

## DISCUSSION

We observed the systemic virus dissemination in all three animal models after intratumoral infusion of adenoviral vectors. In 4T1 tumours, the dissemination occurred mainly during the first 10 min after the infusion was started. The amount of viruses disseminated into the liver could be one order of magnitude higher than that retained in the tumour. The blood and lung in mice contained few transfected cells after the intratumoral infusion. However, tumour cells injected intravenously into the mice accumulated mainly in the lung. These observations in combination with data in the literature allowed us to determine the mechanism of systemic virus dissemination during the first 24 h after the intratumoral infusion was started.

Potentially, the mechanisms of dissemination include cell-mediated transport and diffusion and convection of viral vectors from the interstitial space into the lumen of tumour microvessels. We have showed previously that the diffusion process is too slow to cause the virus dissemination after intratumoral infusion (Wang *et al*, 2003). Based on diffusion, the maximum amount of disseminated viruses would be several orders of magnitude less than that retained in the tumour. This was inconsistent with the observations shown in Figures 1, 2, 4 and 5 and data in the literature (DeWeese *et al*, 2001; Nemunaitis *et al*, 2001; Sauthoff *et al*, 2003). Therefore, diffusion is negligible during the virus dissemination.

The cell-mediated transport was likely to be insignificant as well. The reasons are as follows. First, the amount of adenoviral vectors associated with cells in blood samples shown in Figure 2B was extremely low compared with that in the plasma. Even the cell-associated vectors could be originally in the plasma and then nonspecifically bind to erythrocytes (Cichon *et al*, 2003) or be co-precipitated with blood cells during the centrifuging process. Second, most tumour cells injected intravenously accumulated in the lung (Figure 3), which was consistent with data in the literature that lung is the primary target of metastatic tumour cells in mice (Lee, 1983). Third, few transfected cells existed in the blood and lung at 24 h after the intratumoral infusion (Figure 4). Therefore, the observations shown in Figure 2B indicated that blood-borne cells played an insignificant role in the virus dissemination, while the observations shown in Figures 3 and 4 indicated that the virus dissemination was not mediated by transfected tumour cells either.

Convection is in general negligible in the centre of solid tumours, due to uniformly elevated interstitial fluid pressure and intrinsic leakiness of tumour microvessels (Jain, 1997). Convection is important only during the intratumoral infusion and the first few minutes after the intratumoral infusion when a fluid pressure gradient is created around the tip of the infusion needle. After the infusion, the pressure gradient should decrease rapidly with time since (a) the equilibrium time constant between the microvascular pressure and the interstitial fluid pressure is approximately 10 s (Netti *et al*, 1995) and (b) the microvascular pressure in the tumour should return to the undisturbed level within a few minutes after the infusion, due to blood circulation. Therefore, convection can only cause virus dissemination at early time points but becomes insignificant at late time points.

Taken together, the virus dissemination occurred after the adenoviral vectors entered the system circulation via infusion-induced convective transport. The entrances were the leaky microvessels that were either intrinsically hyperpermeable, with pores in the microvessel wall being larger than adenoviruses (Hobbs *et al*, 1998; Yuan, 1998) or damaged by the infusion procedures. In the blood, adenoviruses were rapidly taken up by Kupffer cells and hepatocytes in the liver, with a plasma half-life of ∼2 min (Alemany *et al*, 2000b). The dissemination of the adenoviral vectors was significant only during the first few minutes (<10 min) after the intratumoral infusion.

The ratio of copy numbers of adenoviruses between liver and tumour shown in Figure 5 had a large variation among different animals. This result was qualitatively consistent with the ratio of bioluminescence intensities between liver and tumour after intratumoral infusion of AdCMVLuc (data not shown). Such variations were unlikely to be caused by the experimental procedures because the tip of the needle had been carefully placed near the centre of all tumours and the infusion protocol had been standardised by using a syringe pump. The variation could be due to the heterogeneous distribution of microvessels, a hallmark of solid tumours. As a result, the number of leaky microvessels involved in virus dissemination would depend on vascularisation and location of the needle tip in tumours.

Systemic virus dissemination after intratumoral infusion has been investigated in previous studies (DeWeese *et al*, 2001; Nemunaitis *et al*, 2001; Sauthoff *et al*, 2003). Sauthoff *et al* (2003) have detected a self-replicating adenoviral vector in mouse blood at 10 min after intratumoral infusion, based on the assay of virus titration on 293 cells. The number of viral DNA copies in the blood is one order of magnitude lower than that retained in the tumour and no adenoviruses can be detected in the liver during the first week after the intratumoral infusion. The liver data are inconsistent with those in the literature, which have demonstrated that nearly 99% of adenoviruses in the systemic circulation will eventually accumulate in the liver of mice (Herz and Gerard, 1993; Hackett *et al*, 2000). This discrepancy is likely to be caused by the inappropriate use of virus titration assay. It is well known that most adenoviruses in the liver are internalised by Kupffer cells or hepatocytes. The internalisation and the subsequent intracellular trafficking of adenoviruses may make these vectors lose their ability to transfect 293 cells. Therefore, a better way to determine the virus accumulation in the liver is to quantify the copy number of unique viral DNA sequences as shown in this study. Sauthoff *et al* (2003) have also shown that the adenoviral vectors can be detected in the blood at 2–8 weeks after the intratumoral infusion, presumably due to the replication of vectors in tumours. We used nonreplicating vectors so that we did not observe virus dissemination at late time points (Wang *et al*, 2003).

In a clinical study, the amount of adenoviral DNA in the blood at 30 min after the intratumoral infusion has varied between 0.007 and 0.129% of that infused into tumours (DeWeese *et al*, 2001). The amount of adenoviruses disseminated into the liver or other normal tissues is not determined in patients. Based on the blood data alone, one cannot estimate the amount of disseminated adenoviruses because it is likely that most adenoviruses disseminated during the intratumoral infusion have already been taken up by the liver at 30 min (Alemany *et al*, 2000b) (see also Figures 2 and 5).

In summary, we demonstrated that the virus dissemination occurred during the first few minutes after the intratumoral infusion was started. It was due to infusion-induced convective transport of viral vectors into leaky tumour microvessels. In future studies, we will investigate effects of infusion rate and pressure as well as structures of tumour tissues on virus dissemination. The findings in this study suggest that it is desirable to develop polymeric delivery systems that can increase the concentration of adenoviruses in solid tumours and simultaneously reduce the amount of disseminated adenoviruses in normal tissues, particularly in the liver, thereby improving the efficacy/toxicity ratio in cancer gene therapy.
